# Hypopituitarism as consequence of late neonatal infection by Group B streptococcus: a case report

**DOI:** 10.11604/pamj.2015.20.308.6538

**Published:** 2015-03-30

**Authors:** Amanda Santana Ferreira, Ana Lourdes Lima Araújo Fernandes, Guilherme Guaragna-Filho

**Affiliations:** 1 Pediatric Emergency Unit, Complexo Hospitalar Prefeito Edivaldo Orsi (Ouro Verde), Campinas, São Paulo, Brazil; 2 Pediatric Nephrologist, Pediatric Clinic Unit, Complexo Hospitalar Prefeito Edivaldo Orsi (Ouro Verde), Campinas, São Paulo, Brazil; 3 Pediatric Endocrinologist, Pediatric Emergency Unit, Complexo Hospitalar Prefeito Edivaldo Orsi (Ouro Verde), Campinas, São Paulo, Brazil

**Keywords:** Hypopituitarism, Group B streptococcus, streptococcus agalactiae, meningitis

## Abstract

Hypopituitarism is a condition characterized by dysfunction of the pituitary gland hormone production. The insults of the perinatal period, which includes the late infection by Group B Streptococcus, consists in a rare etiology of this condition. We present the case of a 39-days-old infant with meningitis caused by Streptococcus Group B, which showed, among other consequences, hypopituitarism.

## Introduction

Hypopituitarism is a condition characterized by dysfunction of the pituitary gland hormone production, thereby failing to achieve the body's demands adequately [1]. Among its various possible etiologies, the insults of the perinatal period are a rare cause [1]. Streptococcus agalactiae or Group B Streptococcus (GBS) is the major cause of neonatal infection in the Western world [2]. Early GBS infection, which occurs in the first 7 days of life, typically presents with pneumonia and septicemia and it is related to the contact with the pathogen in maternal genitourinary tract during labor [2]. However, the late infection occurs between 7 and 90 days and it is characterized by meningitis and / or sepsis [2]. The pathogenesis of this presentation is not well understood, but it seems to be related to the horizontal and vertical transmission, it may have the involvement of breastfeeding, maternal colonization of the pharynx and nosocomial sources [2–6]. Our aim is to report a case of late GBS infection, with meningitis and sepsis as the initial clinical manifestation, which showed, among other consequences, hypopituitarism.

## Patient and observation

**Figure 1:**
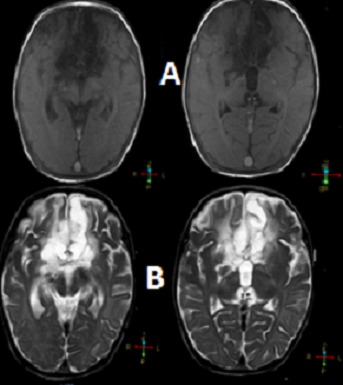
MRI of the patient: (A) Before the contrast impregnation, (B) Images with contrast: highlight for extensive diffuse inflammatory / infectious involvement of the meninges with impaired signals associated underlying brain parenchyma, especially in frontal region, left nucleo-capsular region, midbrain and pons. Subdural collections of thick content, not hemorrhagic, in right frontoparietal region and left frontal region

**Figure 2:**
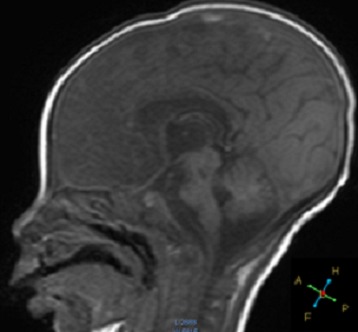
MRI of the patient, sagittal image: it was not possible to identify the Pituitary

Male patient, caucasian, born by normal delivery at term, birth weight of 4145g, birth length of 53.3 cm and head circumference of 42 cm. There was no gestational or neonatal complications. The mother, previously healthy, attended 11 prenatal visits, which began in the seventh week of gestation. During the pregancy, the screening for toxoplasmosis, syphilis, HIV, hepatitis B and hepatitis C were negative and two urine cultures were held (on the 1^st^ and 3^rd^ trimester), both without bacterial growth. When he with 39 days of age, the parents sought for assistance in the emergency room of another municipal hospital in the city of Campinas, state of São Paulo (Brazil) because the patient was presenting poor clinical condition, hypoactive, febrile and hemodynamically unstable. In the initial management, he was placed on mechanical ventilation, performed fluid resuscitation and started dobutamine, norepinephrine and empirical antibiotic therapy with ceftriaxone. The initial complete blood count showed just leukopenia and blood cultures were positive for GBS. Cerebrospinal fluid analysis showed 498 cells with a predominance of neutrophils (96%), glucose consumption (0 mg/dl), protein concentration (467 mg/dl), bacterioscopy with gram positive cocci and culture showing GBS. It was started ampicillin and ceftriaxone was maintained. After stabilization, in approximately 24 hours, he was transferred to the Intensive Care Unit Pediatric of our hospital, where the conduct was maintained (held antibiotic therapy for 10 days). The patient evolved with non-progressive chronic encephalopathy and seizures, which were controlled with phenobarbital and clonazepam. On the second day of admission in our hospital, polyuria was observed, with a urinary volume of 4 liters per day without the use of diuretics. The laboratory tests showed a variable natremia in a period of 24 hours (130-165 mEq/dl), with a predominance of hypernatremia. At that time, it had been tried to correct this by replacing free water, however without success. After 5 days in an attempt to control the electrolyte disturbance, a dose of intranasal desmopressin (2.5mcg/day) has been done and then it was observed a normalization of the natremia as well as urinary concentration and decreased of its total volume. Once it had been suspected of central diabetes insipidus, laboratory tests has been done (Table 1), with the samples collected after the initial use of desmopressin, in order to investigate the whole hypothalamic-hypophysary axis. Based on these results, continuous therapy with desmopressin has been started, with gradual dose increases until the complete resolution of the symptoms and of the laboratory alterations. The tests have identified adrenocortical insufficiency and central hypothyroidism, so it has been initiated replacement therapy with hydrocortisone and levothyroxine, respectively. A failure of the somatotropic axis has been also diagnosed, nevertheless it has been decided to not initiate growth hormone therapy in this first moment. The gonadal axis, due to the age of the patient, was not investigated. A Magnetic Resonance Imaging (MRI) of the central nervous system (CNS) has been done (Figure 1, Figure 2). It was necessary to submit the patient to an elective tracheotomy in order enable the ventilatory weaning, but it has been maintained the need for oxygen therapy. Among other neurologic consequences, the patient has presented swallowing disorders and neurogenic bladder. Thereby, elective gastrostomy and vesicostomy were also performed.

**Table 1:** Diagnostic laboratory data

Tests	Result	Reference values	Method
Serum sodium (mEq/L)	166	(135 - 145)	Potentiometry and selective electrode
Urinary sodium (mEq/L)	26	(40 - 80)	Selective electrode
Serum osmolality (mmol/L)	349	(280 - 300)	Crioscopy
Urinary osmolality (mmol/L)	113	(250 - 900)	Crioscopy
Urinary density	1005	(1005 - 1035)	Physical and chemical
TSH (uUI/L)	6.67	(0.8 - 6.3)	Chemiluminescence
Free Thyroxine (Free T4) (ng/dl)	0.66	(0.7 - 1.90)	Chemiluminescence
IGF-1 (ng/ml)	28	(19 - 109)	Chemiluminescent immunometric
IGFBP-3 (mcg/ml)	1.0	(0.7 - 3.6)	Chemiluminescence
ACTH (pg/ml)	<5.0	(7,2 - 63,3)	Electrochemiluminescence
Cortisol (ng/ml)	3.5	(6,7 - 22,6)	Chemiluminescence

## Discussion

The GBS is a leading cause of neonatal meningitis and sepsis, as well as other serious newborns infections, associated with significant morbidity and mortality [4–6]. A recent meta-analysis has shown an overall average incidence of 0.53 per 1000 live births, with an incidence rate twice of the early form when compared to the late one [7]. When analyzed only the late infection, the African continent with the highest incidence, 0.71, followed by the Americas, these presenting 0.31 per 1000 live births [7]. Furthermore, this meta-analysis found a two-fold higher fatality rate in the early onset GBS infection than in the late form [7]. In the researches focused only in late disease, the incidence ranged from 0.15 to 0.45 per 1000 live births [3, 5]. Moreover, in these studies, meningitis, as the clinical presentation, showed a relationship with severe neurological sequelae [3, 5]. Analyzing the prenatal period, we found that the patient's mother has done a very appropriate gestational follow-up according to the recommendations of the Ministry of Health of Brazil [8]. Nevertheless, this office has no guidelines concerning the screening and prophylaxis of GBS [8]. In this sense, the Centers for Disease Control (CDC) of the United States recommends universally search for the presence of colonization by GBS in pregnant women through vaginal and rectal culture between the 35th and 37th week of gestation [9]. In relation to our patient, apparently, intrapartum prophylaxis would not have prevented the onset of disease, since this measure is to avoid early disease and there is no evidence that it can change the course of late infection [2, 9]. Regarding the endocrine sequelae of GBS meningoencephalitis in newborns, there are few data in the literature. In our review, we found a case report of hypopituitarism secondary to CNS infection by GBS similar to our patient. In this 1976 report, Pai et al described the case of an 1 month-old infant with late infection, which presented a frank diabetes insipidus as the first endocrine manifestation [10]. The adrenocorticotropic and thyrotropic axes of this patient were also evaluated showing insufficiency [10]. In this case, the somatotropic axis was also evaluated and showed failure; however this investigation was done in an older age [10]. In 2010, Ko et al reported the case of a child whose mother had a positive screening for GBS and then it has been done the intrapartum prophylaxis [6]. The patient has received prophylactic antibiotics in the first days of life [6]. However, with 28 days of life, the patient developed a serious GBS infection with severe CNS involvement [6]. The authors report hypothalamic dysfunction, but no details were described [6]. Finally, we highlight that our patient has already a less favorable prognosis due to his consequent chronic encephalopathy. In addition to this, it has been reported that patients with hypopituitarism have an increased mortality rate associated with respiratory, cardiovascular and cerebrovascular causes [1, 11]. The latter is related, among other factors, with the hormonal issue [1, 11].

## Conclusion

The late neonatal GBS infection and its consequences still have an epidemiological relevance especially in Africa and the Americas. In this context, the hypopituitarism, which may arises as a consequence of a serious CNS injury, contributes to the detriment of the patient's prognosis.
